# Biosynthesis of fluorescent silver nanoparticles from *Leea coccinea* leaves and their antibacterial potentialities against *Xanthomonas phaseoli* pv *phaseoli*

**DOI:** 10.1186/s40643-020-00354-2

**Published:** 2021-01-04

**Authors:** María del Carmen Travieso Novelles, Annie Rubio Ortega, Beatriz Alvarez Pita, Mylene Corzo López, Lianet Díaz Pérez, Emilio Acosta Medina, Oriela Pino Pérez

**Affiliations:** 1 Laboratory of Chemical-Ecology, National Center for Animal and Plant Health (CENSA), San José de las Lajas, Mayabeque Cuba; 2 Laboratory of Plant Bacteriology, National Center for Animal and Plant Health (CENSA), San José de las Lajas, Mayabeque Cuba; 3 Center of Advanced Studies of Cuba (CEA), La Habana, Cuba

**Keywords:** Leaves extract, Silver nanoparticles, Antibacterial properties, Xanthomonas

## Abstract

The synthesis of silver nanoparticles (SNP) from plants is a simple, fast and environmentally safe route. In the present study, the aqueous extract of fresh leaves from *Leea coccinea* L. was evaluated as a possible source of reducing and stabilizing agents to obtain SNP. The synthesized SNP were characterized by spectroscopic techniques such as UV–visible spectrophotometry and Fourier transform infrared spectroscopy (FTIR), scanning electron and confocal microscopies and the antimicrobial activity against *Xanthomonas phaseoli* pv. *phaseoli* was evaluated using agar diffusion methods. The results showed that the evaluated extract was promising for the green synthesis of the SNP, which was visually identified by the formation of a dark-brown complex and the presence of a peak of maximum absorption at 470 nm in a UV–VIS spectrum. FTIR spectrum of SNP showed main characteristic signals of aromatic compounds, carboxylic group among others confirmed by phytochemical screening that made evident the presence of flavonoids, phenols, leucoanthocyanidins, terpenes and steroids groups. Fluorescent SNP with high degree of agglomeration were observed by the microscopical technics used. A promising antibacterial activity of SNP was shown by a zone of microbial growth inhibition. These results suggested the need for going deeper in the physico-chemical characterization and kinetic studies, as well as the biological evaluations to make possible the use of this plant source in the future development of antibacterial formulations for bean seed protection.
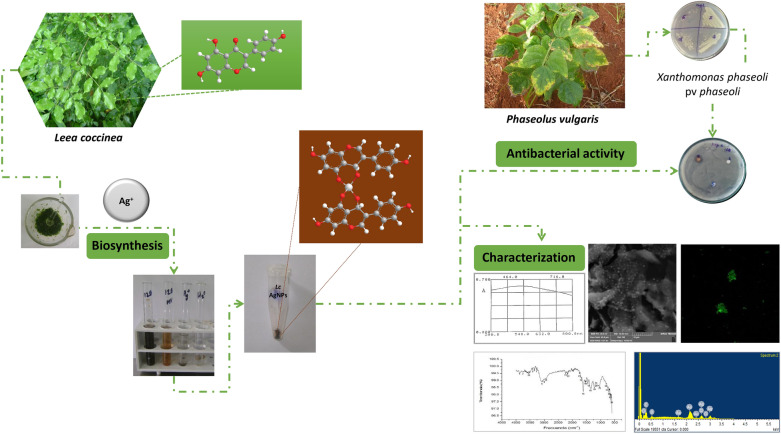

## Introduction

Phytopathogenic bacteria cause a negative impact on agricultural production systems, decrease yields and increase economic losses (Pedroza et al. 2013). The genus *Xanthomonas* (Proteobacteria) is a diverse group of Gram-negative bacteria that affects numerous crops (Pedroza et al. 2013; Corzo et al. 2015). The disease known as common bacteriosis or common blight, widely distributed in the world, is caused by *Xanthomonas phaseoli* pv. *phaseoli* (Smith) Vauterin and *X. citri* subsp. *fuscans* (Burkholder) Starr & Burkholder (Corzo et al. 2015). This disease is the main biotic factor affecting the yields of *Phaseolus vulgaris* (common bean) causing losses in this crop of 10–40%, depending on the cultivar susceptibility and environmental conditions (Pedroza et al. 2013; Corzo et al. 2015; Constantin et al. 2016; Francisco et al. 2013).

At present, management of this pest is complex and multifactorial, in which the seed control is essential. In this sense, efforts are made for searching resistant varieties (Rodríguez et al. 2015), biological and chemical controls (Aguilar et al. 2019; Ararsa et al. 2018) as a means to guarantee its quality. Numerous authors point out that the foliar application of copper-based bactericides, crop rotation, intercropping, among other actions, are successful if they are applied in an integrated management program (Ararsa et al. 2018). The use of chemical pesticides based on copper, either for the treatment of the seed or systemic treatment, is one of the most widespread alternatives to control this pest; however, numerous studies have shown the resistance of the bacteria (Ram et al. 2018).

In recent years, there has been an increase in research focused on developing nanotechnologies based on the potentialities of biological systems (microorganisms, algae, plants, etc.) to endogenous production or extracellular secretion of metabolites capable of, by different types of reactions (e.g., redox with metallic elements), form coordination complexes of particle sizes in the order of nanoscale, with potential applications in dissimilar fields, including agriculture (Rajesh et al. 2012; Shende et al. 2016). Plants are the most ancestral and fruitful source for obtaining active substances, however, many authors recognize the infinite arsenal of these botanical sources of which only a small fraction has been studied within the great diversity available globally (De Luca et al. 2012; Palhares et al. 2015; Newman and Cragg 2016; Rai et al. 2009). One aspect of pharmacognostic studies is the synthesis of biogenic nanoparticles from the reaction of phytochemical compounds present in extracts of plant parts with metal cations (e.g., Ag^+^) or other substances yielding nanoparticles (NPs) which in their structure contain the reduced metallic cation covalently bound to the phytochemical compounds or secondary metabolites (alkaloids, flavonoids, phenolic compounds, polysaccharides, vitamins, amino acids, terpenoids, etc.), which drastically change the physical–chemical properties (optical, electrical, magnetic, etc.) and biological effects of the metals and compounds involved (Rai et al. 2009) with important biological effects at cellular level (Kasithevar et al. 2017). The synthesis of silver nanoparticles (SNP) from plants represents an advantageous technological alternative due to its simplicity, low cost, the possibility of carrying out large-scale productions, as well as involving environmentally friendly processes to obtain a new generation of antimicrobial compounds (Kasithevar et al. 2017; Rao et al. 2016; Sarkar and Goutam 2017; Arokiyaraj et al. 2017; Singh et al. 2014; Sun et al. 2014; Rajathi and Sridhar 2013; Chandrappa et al. 2017; Ronavari et al. 2017; Sharma et al. 2015). Numerous botanical species have been reported for these purposes (Dhand et al. 2016; Suparna and Anantharaman 2017; Kumar et al. 2017; Ibraheem et al. 2016; Anandalakshmi and Venugobal 2017; Algebaly et al. 2020; Travieso et al. 2018; Gupta and Chauhan 2017).

*Leea coccinea* is within the 70 species of the genus Leea belonging to Vitaceae family (before Leeaceae) (International Seed Testing Association 2013) and shows a wide global distribution (Australia, New Guinea, South and Southeast Asia, parts of Africa and the Americas). In Cuba, *L. coccinea* specie abounds in the western region and it is used primarily as a living fence and as an ornamental plant due to the beauty of its foliage and inflorescences. Although other species of the genus have been studied (Emran et al. 2012; Joshi et al. 2016; Mahmud et al. 2017; Halder et al. 2018), reports on the chemical composition of *L. coccinea* are hardly found in the literature reviewed. The antimicrobial activity of Leea species has been demonstrated (Joshi et al. 2016; Tun et al. 2019). Recent reports showed the antinociceptive and anthelmintic activity of *Leea aequata* leaf extracts (Halder et al. 2018). Other authors have justified the antioxidant and antibacterial potential of root tubers of *Leea macrophylla* which is a potential tool in the treatment of disorders associated with oxidative stress and pathogenic infections (Joshi et al. 2016; Mahmud et al. 2017).

The phytochemical screening of ethanolic extract of root tubers of *Leea macrophylla* revealed that it is rich in phenolic compounds (Joshi et al. 2016; Mahmud et al. 2017); saponins (Joshi et al. 2016). Also this extract is rich in carbohydrates between others phytochemical (alkaloids and flavonoids) of high biological value (Joshi et al. 2016).

The aims of the current study were to evaluate the aqueous extract of the *Leea coccinea* leaves, as a possible source of reducing and stabilizing agents for obtaining SNP, as well as to determine its potential as an antibacterial agent against *X. phaseoli* pv. *phaseoli*.

## Materials and methods

### Plant material

**Fig. 1:**
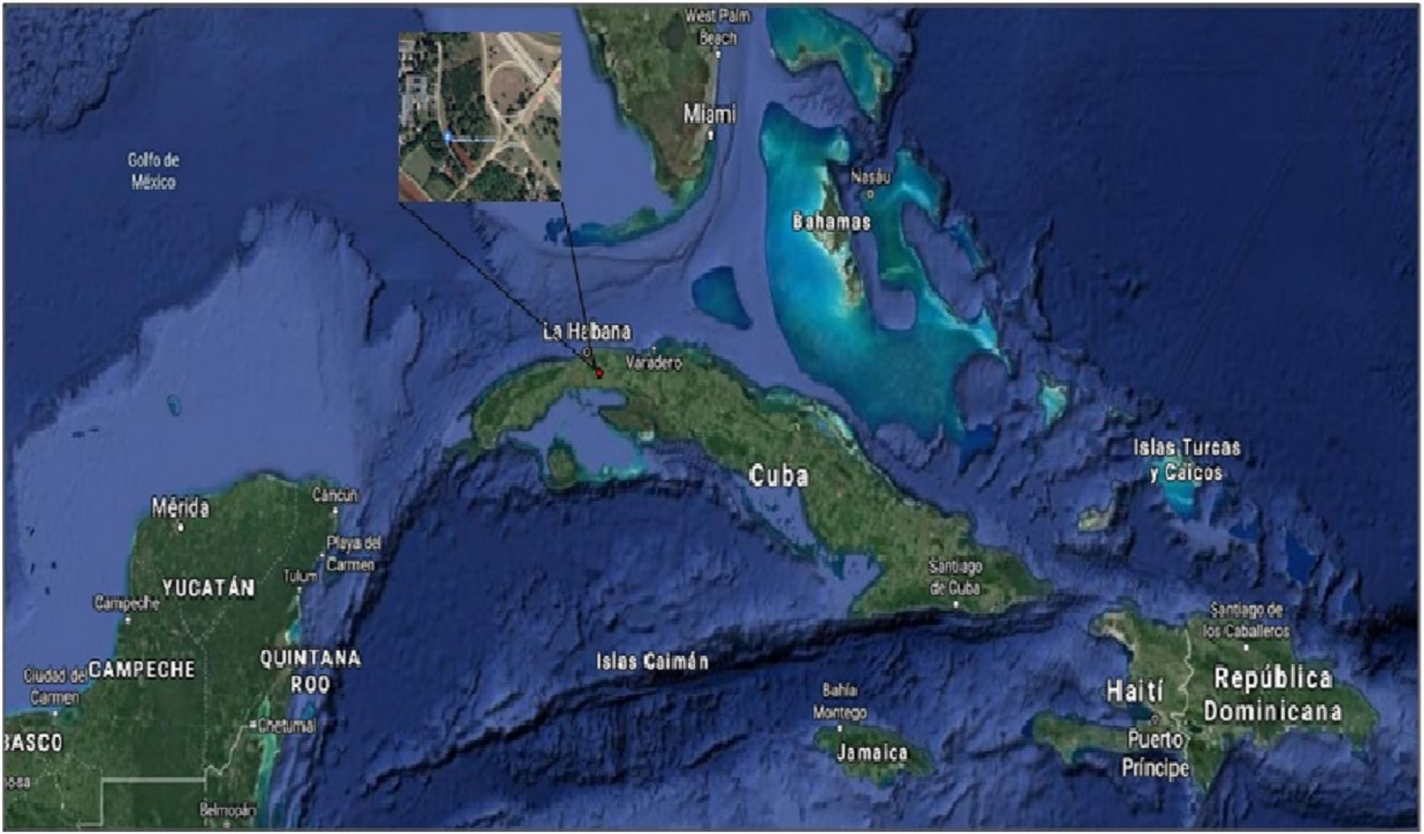
Geo-localization of the plant material of *Leea coccinea*

Leaves of *Leea coccinea* plants, located at 22,990,173 N and − 82, 151,213 W at a height of 142 masl (red point in Fig. 1), were collected and selected to remove soil contaminant. Their authentication was carried out by the specialist in botany from Agrarian University of Havana (UNAH), Mayabeque, Cuba.

### Preparation of plant extracts and biosynthesis of SNP

The collected fresh leaves of *L. coccinea*, thoroughly checked for being free of any contaminant, were triturated to a thick powder. Then 5 g of the powder were macerated with deionized water (1:8) (W/V). This mixture was heated to 90 °C for 10 min, and after being cooled, filtered through Whatman No. 1 paper.

**Fig. 2:**
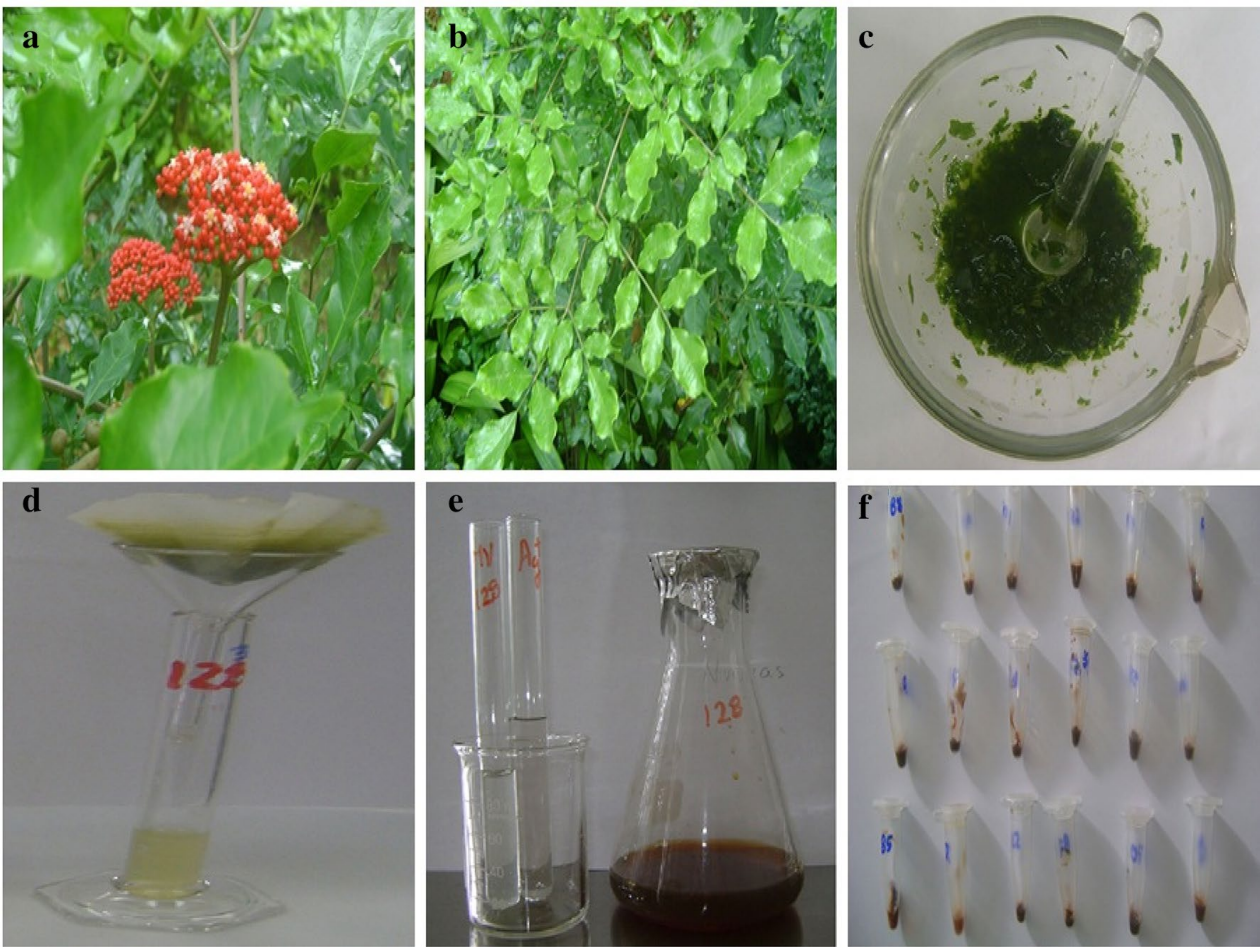
Biosynthesis of SNP from *Leea coccinea* (*Lc*) leaves. **a** Shrubs of *Lc* with flowers; **b** leaves of *Lc*; **c** maceration; **d** filtration; **e** reaction of bio-reduction con Ag^+^; **f** precipitation

SNP synthesis was carried out by a typical procedure (Fig. 2). The solution of silver nitrate (2 mM) was added to the plant extract (9:1) (V/V). It was homogenized with a mechanical stirrer (IKA-VF2) and left to rest for 24 h at room temperature (27 ± 2 °C). The bio-reduction reaction was monitored by visual observation at different times (5 min, 1 h and 24 h). A change of color to dark brown was indicative of the formation of SNP. The resulting suspension of SNP was centrifuged at 10,000 rpm for 20 min, and the pellet was washed with sterile water three times. SNP were stored at room temperature in the dark until evaluation. The yield of SNP was determined by measuring of the dry weight of 1 mL of the suspension for triplicate on an infrared balance (Sartorius MA35) and calculated with relation to the initial mass of plant material. The results were expressed as the media valor in the range of the standard deviation.

### Characterization of SNP

The biosynthesized SNP were characterized by various instrumental analyses. The pH of the aqueous suspension of nanoparticles was determined at different times (5, 10, 30, 60, 90 and 120 min) by a Mettler Toledo instrument. The formation of SNP was demonstrated by UV–VIS spectrum (300–800 nm) through RAYLEIGH (VIS-723G) spectrophotometer and the spectrum of Fourier transform infrared spectroscopy (FTIR) in the range 4500–200 nm by using an IR Prestige 21 equipment (SHIMADZU). The scanning electron microscopic (SEM) analysis was performed using the MIRA 3 LMU (TESCAN) SEM machine. Samples to SEM analysis were applied on carbon adhesive films and coated with gold (5 nm) with a Desk Sputter Coater DSR1. The films were dried during 24 h. The EDX analysis of SNP was carried out on samples using Energy Disperse Ray X (Oxford Instrument). Fluorescence properties were demonstrated with confocal microscopy (FICT-wide 479 nm Lent 20×). It was performed on samples previously sonicated for 10 min and applied on porous albumin slides covered with coverslips, which were kept in a humid chamber until observation.

Phytochemical screening of aqueous extract of leaves of *L. coccinea* was done to identify the presence of secondary metabolites with reducing properties. Several qualitative tests included in the internal protocol established in the Chemical- Ecology Laboratory, and classics reactions such as ninhydrin (free amino acids), ferric chloride (phenols), gelatin (phenols), Liebermann–Burchard (triterpenes and steroids), Kedde (lactonic rings), Borntrager (quinone), Shinoda (flavonoids), Rosenheim (leucoanthocyanidins) and Mayer–Dragendorff–Hager–Wagner to alkaloids, were carried out.

### Evaluation of antimicrobial activity

To evaluate the antimicrobial activity of SNP, the colloidal suspension was precipitated by centrifugation (10,000 rpm for 20 min) to remove excess of salt and others soluble compounds. The silver colloids were washed at least three times with deionized water. The antibacterial activity on *Xanthomonas phaseoli* pv. *phaseoli* was evaluated by the disk agar diffusion method (Clinical and Laboratory d Institute 2015). An inoculum was prepared at the concentration of 29 × 10^7^ UFC mL^−1^, in sodium chloride solution (0.9%), from a culture of 24-h incubation at the temperature of 28 °C in nutrient agar plates, according to the McFarland scale. The strain used was Xap1, belonging to the ceparium of the Laboratory of Plant Bacteriology of the National Center for animal and Plant Health in Cuba (Corzo et al. 2015). For application on disks, 10 µL of the precipitated SNP was applied in every disk. As positive controls, erythromycin (5 µg) and CHLORAMPHENICOL (10 µg) disks were applied. Sterile water was used as a negative control. At least four replications of the treatment and controls were performed. Plates were incubated at 28 °C for 24 h. After this time, zone of inhibition (mm) of bacterial growth were measured with a graduated rule.

The data obtained were statistically processed by a simple variance analysis and the means were compared using the Duncan multiple range comparison test with a significance level of 5%, using the statistical package InfoStat/ L (Di Rienzo et al. 2016).

## Results and discussion

**Fig. 3:**
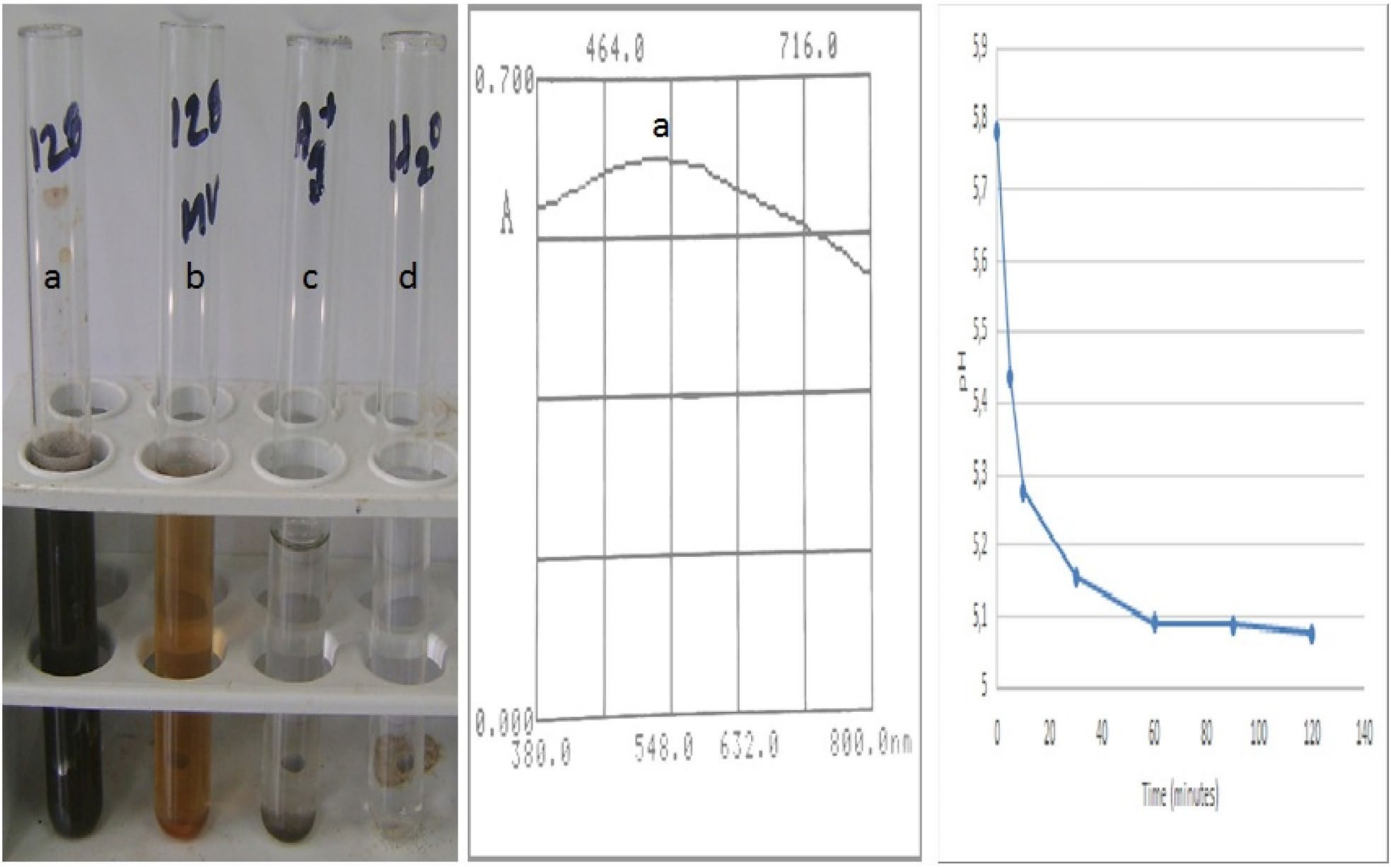
Identification of the formation of SNP from *Leea coccinea*. Visual (**a**), spectrophotometric (**b**) and pH variation during bio-reduction (**c**). a: positive reaction between aqueous extract of leaves (AEL): silver solution 2 mM (1:9) (V/V); b: negative reaction of AEL: distilled water (1:9); c: silver solution 2 mM: distilled water (9:1); d: distilled water

The reaction of bio-reduction between the Ag^+^ cations and the phytochemical compounds of the aqueous extract of *L. coccinea* leaves occurred instantaneously (before 5 min), made evident by the presence of a dark-brown colored complex (Fig. 3a), indicative of the formation of nanoparticles (Vadlapudi and Amanchy 2017; Sarkar and Goutam 2017; Khan et al. 2018). UV–visible spectroscopic absorbance (Fig. 3b) showed a signal with λ_max_ around of 470 nm due to strong surface plasmon resonance, which reaffirms the formation of SNP (Travieso et al. 2018). The release of H^+^ ions since the first minutes of the reaction was verified by the gradual decrease of the aqueous medium pH (Fig. 3c). Some authors have explained the oxidation process of Ag^+^ by phytochemical compounds from plants, which are accompanied by the release of H^+^ ions (Zuorro et al. 2019). Also, it has been proposed that tautomer transformations of other compounds from plants (e.g., flavonoids) from the enol form into the keto form can release reactive hydrogen atoms that reduce metal ions to coordination complex of Ag forming nanoparticles. Nevertheless, due to the complex composition of natural extracts, up to now, there is not enough evidence about which specific phytochemicals are responsible for the formation of nanoparticles (Jeevanandam et al. 2016).

**Fig. 4:**
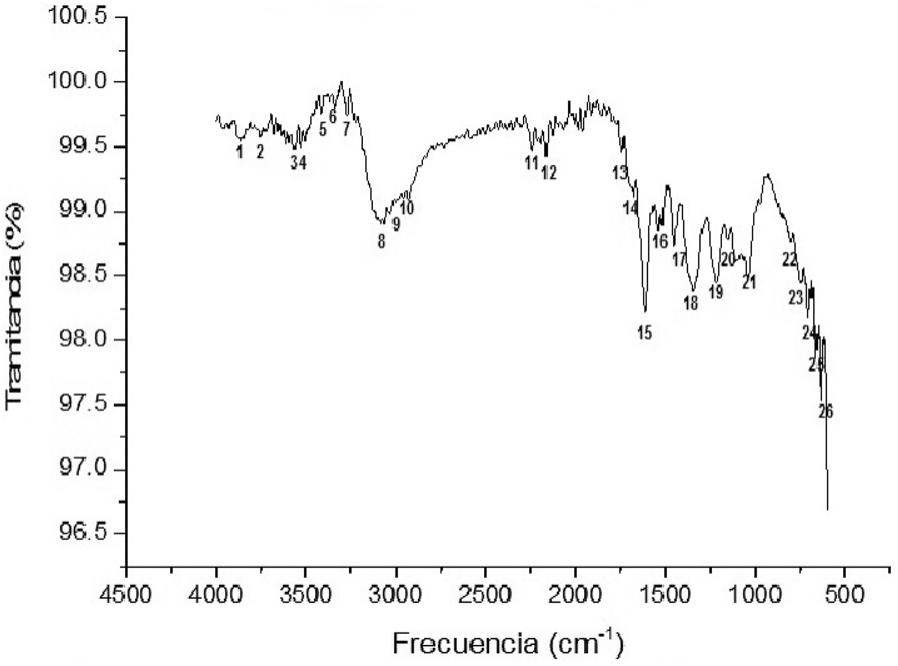
FTIR spectra of SNP synthesized of *L. coccinea* leaf extract. Main peaks: 8 (16, 18) (22–26): aromatic (benzene with substitutions); 15(19): –COOH carboxylic (C=O); 17(10): C=H (methylene)

FTIR spectrum confirmed the phytochemicals screening (Fig. 4). Measurements were carried out to identify the functional groups responsible for the reduction of the Ag^+^ ions and capping of the bio-reduced silver nanoparticles synthesized by *L. coccinea* leaf extract. FTIR spectrum of SNP showed the presence of signals such as strong peaks between 3083 and 3076 cm^−^1 (peak 8) with a signal at 1538 cm^−1^ (peak 16) like aromatic with multiple substitutions (C=C stretching); other peaks were around 1616 cm^−1^ (peak 15) (C=O stretching) of carboxylic group. The absorption at about 1334 cm^−1^ (peak 18) indicates a residual amount of (NO_3_)^−1^ ion in the solution. Others signals appear at 1207 cm^−1^ (peak 19), between 3881 and 3831 cm^−1^ (peak 1), 3752 cm^−1^ (peak 2), 3565 cm^−1^ (peak 3), 3529 cm^−1^ (peak 4), between 3414 and 3335 cm^−1^ (peak 6) suggesting polyphenolic OH group along with the peak around 880 cm^−1^ which represents the aromatic ring C–H vibrations. Others peaks around of 3270 cm^−1^(peak 7), 3025 cm^−1^ (peak 9), between 2932 and 2925 cm^−1^ (peak 10), 2235 cm^−1^ (peak 11), 2171 cm^−1^(peak 12), 1747 cm^−1^ (peak 13), 1688 cm^−1^ (peak 14), 1451 cm^−1^ (peak 17), 1156 cm^−1^ (peak 20) and between 1107 and 1035 cm^−1^ (peak 21) indicating the complexity of samples containing nanoparticles from natural sources and suggesting the need for continuous studies about the chemical structures of compounds involved in bio-reduction.

**Fig. 5:**
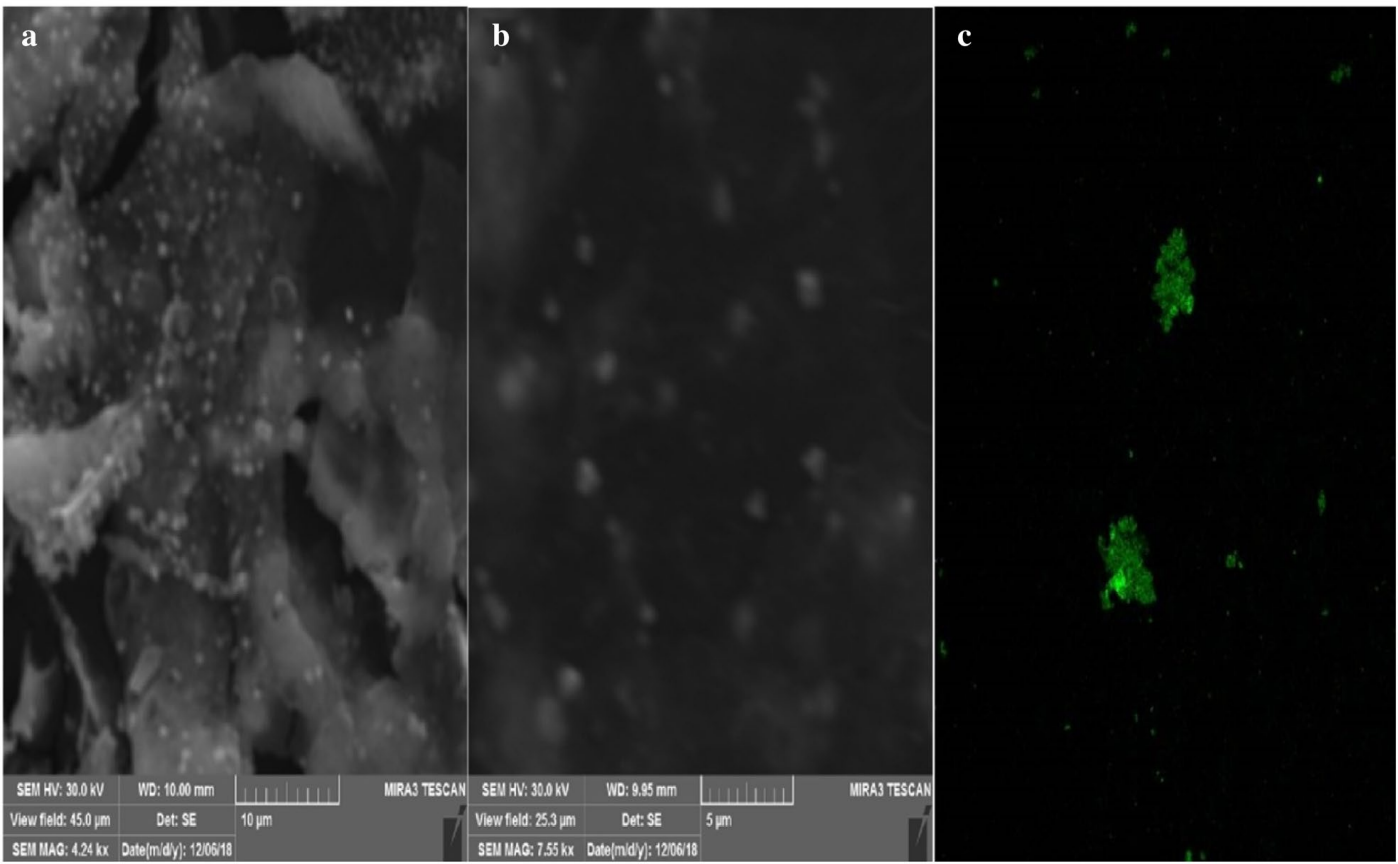
SEM (**a**, **b**) and confocal (**c**) images of the SNP synthesized from *Leea coccinea* leaf extract

The SEM images (Fig. 5a, b) of SNP showed the presence of abundant nanoparticles with high grade of aggregation which was confirmed with confocal microscopy images, where the emitted fluorescence was detected at 479 nm, demonstrating the occurrence of overlapping of smaller particles (Fig. 5c). Numerous authors have reported an agglomeration due to strong electrostatic interactions of metal ions. The fluorescent properties suggest the presence of phytoconstituents or antioxidants with fluorophore groups. Others metal nanoparticles synthesized from plants have been reported with fluorescent properties (Talamond et al. 2015; Donaldson and Williams 2018).

**Fig. 6:**
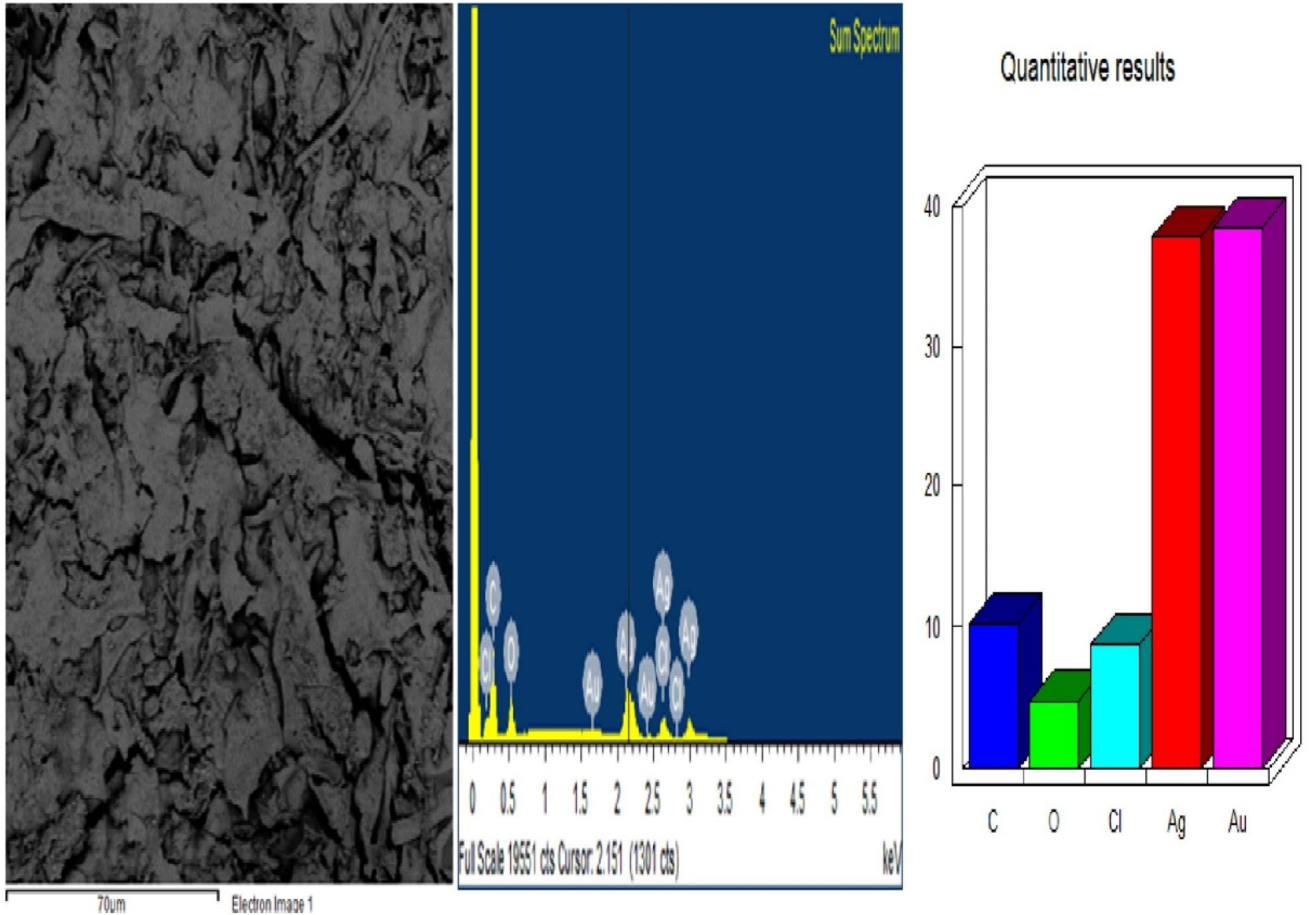
EDX of the SNP synthesized by *Leea coccinea* leaf extract

The results of the qualitative analysis by EDX (Fig. 6) showed a high percentage of Ag made evident by the strong signals in the range of 2–4 keV, as well as the presence of carbon and oxygen indicative of hybrid NPs, with an organic component (e.g., flavonoid) covalently bound to the metallic element. Likewise, the high compaction shows its crystalline nature due to the reduction of Ag^+^ cations by the reducing compounds present in the aqueous extract of *L. coccinea* leaves (Singh et al. 2019; Kasithevar et al. 2017; Moodley et al. 2018; Oluwasogo et al. 2019).

Phytochemical screening showed the presence of secondary metabolites such as phenols, triterpenes and steroids, flavonoids, free amino acids and leucoanthocyanidin in the aqueous extract of leaves of *Leea coccinea* (Table 1). Some of these compounds could react with Ag^+^ to form coordination complexes, so that, due to the presence of several functional groups with reducing properties, kinetic and chemical studies should be carrying out to enlighten the reaction of bio-reduction. At present, very few reports are available on *L.* coccinea phytochemistry. Previous studies on chemical compositions of others species of the genus Leea are in agreement with our previous results. Flavonoids and flavonoid glycosides are found to be the major constituents of the genus (Lakornwong et al. 2014). Chemical studies of *Leea indica* reported six phenolic compounds, flavan-3-ols, flavonoids/flavonoid glycosides, dihydrochalcones and dimeric catechins (Singh et al. 2019). Similar studies based on NMR, MS, and ECD spectra reported one new lignan, one new lactam, five known lignans, four flavonoid glycosides, among other compounds isolated from the ethanol extracts of the aerial parts of *Leea aequata* (Tun et al. 2019).

**Table 1:** Phytochemical screening of the aqueous extracts of *Leea coccinea* leaves

No.	Phytochemical	Test	Result	Qualitative reaction
1	Flavonoids	Shinode	+	
2	Triterpenes and steroids	Liebermann–Burchard	+	
3	Saponins	Froth	−	−
4	Alkaloids	Mayer (M) Dragendorff (D) Hager (H) Wagner (W)	− − − −	
5	Phenols	Ferric chloride Gelatin	+ +	
6	Lactonic rings	Kedde	−	
7	Free amino acids	Ninhydrin	+	
8	Leucoanthocyanidins	Rosenheim	+	
9	Quinones	Borntrager	−	

- absent; + present

**Fig. 7:**
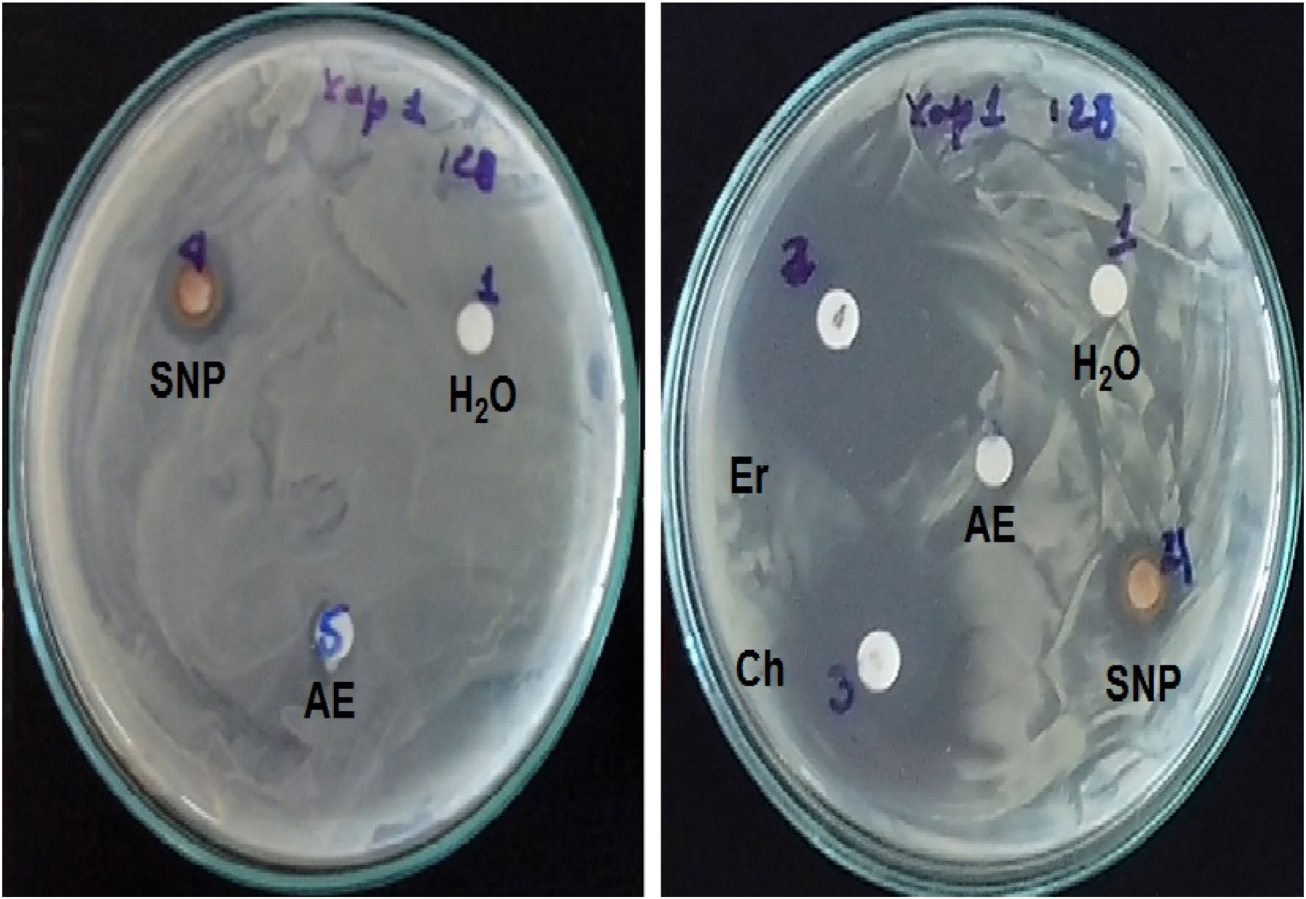
Antibacterial activity of SNP from *Leea coccinea* leaves against *Xanthomonas phaseoli* pv *phaseoli*. SNP: silver nanoparticles; H_2_O: sterile water; Er: erythromycin; Ch: chloramphenicol; AE: aqueous extract of fresh leaves of *L. coccinea*

SNP showed a promising activity against the tested Gram-negative bacterial strain of *X. phaseoli* pv *phaseoli* (Fig. 7, Table 2). In previous studies where the activity of AgNPs synthesized from the residual extract of the hydrodistillation of the essential oil of *Thymus vulgaris* was evaluated, similar activity against this bacterium (Travieso et al. 2018) was demonstrated. However, in the present study, in which the precipitated NPs were evaluated, little diffusion of these nanostructures was found, so biological evaluation studies must be completed with the addition of stabilizing agents. On the other hand, the antimicrobial activity of AgNPs depends on parameters such as Ag concentration, as well as on the size and shape of the nanostructures (Duval et al. 2019). However, other parameters have recently been shown to influence antibacterial activity such as stabilization in the aqueous medium and the accessibility of the surface of bacteria cell by NPs (Duval et al. 2019).

**Table 2:** Antibacterial activity of SNP synthesized from aqueous leaf extract of *L. coccinea* against *Xanthomonas phaseoli* pv *phaseoli*

Number	Sample	*n*	Zone of inhibition (mm)
1	Sterile water (H_2_O)	8	NI
2	Erythromycin (Er)	4	33,00 ± 1,41 *a*
3	Chloramphenicol (Ch)	4	29,00 ± 1,41 *b*
4	SNP	8	12,00 ± 0,00 *c*
5	Aqueous extract (AE)	4	NI

Values ± SD indicates the replicates of the experiment (n)

Formation of aggregates and agglomerates are the main destabilizing factor. They reduce the accessible surface area in contact with the bacterial cell and also inhibit the diffusion capacity in the in vitro tests (diffusion in agar), interfering with the results. The diffusion coefficients of the silver nanoparticles are generally related to size and physico-chemical characteristics (Xiaoxue et al. 2020). For this reason, diffusion methods are used in initial or screening studies; however, they are not valid for the determination of minimum inhibitory concentrations (MIC) and minimum bactericidal concentrations (MBC), suggesting tests such as serial dilutions in which, in addition, the liquid medium facilitates the interaction of the substances to be evaluated with the microorganism through agitation.

Limitations of the antimicrobial activity studies of AgNPs have been pointed out by some authors, suggesting, among other standardized ones, those methods with a liquid medium (macrodilution and microdilution), the Kirby–Bauer method, prior knowledge of the diffusion coefficient of the sample.

## Conclusions

The present research reports, for first time, the biosynthesis of fluorescent silver nanoparticles from aqueous extract of *L. coccinea* fresh leaves through of a simple, fast, economically feasible and environmentally safe route. SNP formation was confirmed visual and spectroscopically (UV–VIS and FTIR spectrum), and its high degree of agglomeration and fluorescence were visualized by the microscopic techniques used. Several functional groups characteristic of flavonoids, phenols, leucoanthocyanidins, terpenes and steroids with reducing properties could be involved in the cationic bio-reduction. The antibacterial activity against *X. phaseoli* pv *phaseoli* of the purified SNP was demonstrated by a zone of inhibition of the microbial growing. Sustainable use of this natural resource constitutes an advantageous alternative for this purpose. Future research should be directed to chemical, biological and pharmaceutical studies for the future design of stable, effective and safe formulations for plant protection.

## Data Availability

All data generated or analyzed during this study are included in this article.

## Abbreviations

SNP: Silver nanoparticles
masl: Meters above sea level
FTIR: Fourier transform infrared spectroscopy
UV–VIS: Ultraviolet–visible
nm: Nanometer
NP: Nanoparticle
MNP: Metallic nanoparticle
W/V: Weight/volume
SEM: Scanning electron microscopy
EDX: Energy dispersive X-ray
NMR: Nuclear magnetic resonance
MS: Mass spectrometry
ECD: Electronic circular dichroism
